# The association between disruption of the circadian rhythm and aggravation of colitis in mice

**DOI:** 10.1093/gastro/goac028

**Published:** 2022-06-16

**Authors:** Yi-Dong Chen, Rui-Feng Zhao, Gen Zheng, Fang-Mei Ling, Jun-Rong Li, Ming-Yang Xu, Di Guo, Qiu-Lei Zhang, Shuang Li, Liang-Ru Zhu

**Affiliations:** Division of Gastroenterology, Union Hospital, Tongji Medical College, Huazhong University of Science and Technology, Wuhan, Hubei, P. R. China; Division of Gastroenterology, Union Hospital, Tongji Medical College, Huazhong University of Science and Technology, Wuhan, Hubei, P. R. China; Division of Gastroenterology, Union Hospital, Tongji Medical College, Huazhong University of Science and Technology, Wuhan, Hubei, P. R. China; Division of Gastroenterology, Union Hospital, Tongji Medical College, Huazhong University of Science and Technology, Wuhan, Hubei, P. R. China; Division of Gastroenterology, Union Hospital, Tongji Medical College, Huazhong University of Science and Technology, Wuhan, Hubei, P. R. China; Division of Gastroenterology, Union Hospital, Tongji Medical College, Huazhong University of Science and Technology, Wuhan, Hubei, P. R. China; Division of Gastroenterology, Union Hospital, Tongji Medical College, Huazhong University of Science and Technology, Wuhan, Hubei, P. R. China; Division of Gastroenterology, Union Hospital, Tongji Medical College, Huazhong University of Science and Technology, Wuhan, Hubei, P. R. China; Division of Gastroenterology, Union Hospital, Tongji Medical College, Huazhong University of Science and Technology, Wuhan, Hubei, P. R. China; Division of Gastroenterology, Union Hospital, Tongji Medical College, Huazhong University of Science and Technology, Wuhan, Hubei, P. R. China

**Keywords:** circadian rhythm, mitochondrial energy metabolism, *PER2*, colitis, inflammatory bowel disease

## Abstract

Delayed recovery from ulcerative colitis is mainly due to impaired healing of the intestinal epithelium after inflammation. The circadian rhythm controls cell proliferation and energy metabolism. However, the role of circadian genes in inflammatory bowel disease is largely unknown. The purpose of this study was to investigate whether disrupting the circadian rhythm in mice can worsen colitis by altering mitochondrial energy metabolism. Mice in the experimental groups were under physiologic stress with an 8-h light shift jet-lag schedule every 3 days, whereas those in the control group were not. Subsequently, half of the mice in the control and jet-lagged groups were given dextran sodium sulfate (DSS) to induce colitis. Mice in each group were euthanized at zeitgeber time (ZT)0, ZT4, ZT8, ZT12, ZT16, and ZT20. To investigate the effects of jet lag on the mice, colon specimens were subjected to hematoxylin and eosin staining to analyse mRNA and protein expression of core circadian clock genes (*Bmal1*, *Clock*, *Per1*, *Per2*, *Cry1*, *Cry2*, and *Nr1d1*). We analysed the mitochondrial morphology, adenosine triphosphate (ATP) levels, and the expression of dynamin-related protein 1 (Drp1) and ser637-phosphorylated (p)-Drp1, which are closely related to ATP production. We further investigated the effect of *PER2* knock-down in the colon epithelial cells (CCD 841 CoN) by measuring ATP and cell proliferation levels. Disrupting the circadian rhythm changed the oscillation of clock genes in the colon of mice, altered the mitochondrial morphology of the colon specimens, decreased the expression of p-Drp1, reduced ATP production, and exacerbated inflammatory responses in mice with DSS-induced colitis. Additionally, silencing of *PER2* in the colon epithelial cells reduced ATP production and cell proliferation. Disrupting the circadian rhythm in mice decreases mitochondrial energy metabolism in the colon and exacerbates symptoms of colitis.

## Introduction

Crohn's disease and ulcerative colitis are two major types of inflammatory bowel disease (IBD), which is a chronic and non-specific inflammatory disease of the intestine with an unknown etiology that is often hard to treat. Crohn's disease affects the whole digestive tract, but mainly the distal ileum and colon, and is characterized by intestinal transmural inflammation, jumping lesions, and segmental longitudinal ulcers that lead to complications such as fistulas, stenosis, and fibrosis [1]. Ulcerative colitis is a diffuse inflammation of the superficial mucosa in the rectum that extends to the distal colon. Symptoms typically include abdominal pain, diarrhea, and the presence of blood, mucus, or pus in the stool [2]. The destruction of the epithelial barrier and its poor regeneration are critical factors that make ulcerative colitis difficult to treat [3, 4].

The circadian clock is a biological mechanism that controls the circadian rhythm to ensure synchronization of internal physiology with the external environment [5, 6]. In mammals, the principal circadian pacemaker is in the bilateral suprachiasmatic nuclei of the hypothalamus [7]. The retina projects information about environmental light, which is the main zeitgeber, to the suprachiasmatic nuclei to correct for deviations in the period and phase of the endogenous clock. This central pacemaker dictates the stage of other oscillators residing in different brain regions and peripheral tissues via endocrine or neural connections [8–10]. The transcription–translation feedback loop, a negative feedback loop of the circadian clock, is present in most tissues. The circadian clock is closely linked to metabolism, growth, cancer, and immunity [11]. Therefore, the main genes associated with the circadian clock (*Clock*, *Bmal1*, *Pers*, *Crys*, and *Nr1d1*) play an essential role in regulating many physiologic processes such as macronutrient absorption [12], intestinal epithelial cell proliferation [13], colon motility [14], and mucus secretion in the intestines [15].

Mitochondria are dynamic organelles that continuously adapt to the energy needs of cells through fission and fusion [16]. The morphology of mitochondria is closely related to their biological function and elongated mitochondria are thought to produce more adenosine triphosphate (ATP) [17]. During the initial stages of cell apoptosis, the balance of mitochondrial fusion and fission is disrupted resulting in mitochondrial fragmentation [17]. Mitochondrial ATP production is linked to the circadian rhythm as Drp1 is involved in the process of mitochondrial fusion and fission. Schmitt *et al*. [18] observed recently that the amount of Drp1 expressed within 24 h is constant. Serine 637(p-Drp1) is what regulates the diurnal variation of ATP production; the expression of Per2 is positively correlated with p-Drp1 [18], thus energy metabolism is under the control of circadian genes. The circadian clock has been shown to maintain the functioning of the heart [19], skeletal muscles [20], pancreatic β cells [21], and the liver by regulating mitochondrial metabolism [22]. However, association between the circadian clock and mitochondria in the colon has not been reported.

Previous study has confirmed that the tight link between the circadian clock and the immune system affects the occurrence and development of IBD [23]. The epithelium is the surface barrier of the intestine and the central immune regulator of the intestinal mucosa [24]. Dysfunction of this epithelial barrier plays a significant role in the etiology and pathology of IBD [25]. Little research has been done on the relationship between the circadian rhythm, mitochondrial metabolism, and epithelial regeneration in IBD.

Our study examined potential associations between the circadian clock and mitochondrial metabolism in the colon tissue of mice. We established a model with jet-lagged vs non-jet-lagged mice and used dextran sodium sulfate (DSS) to induce colitis in the mice. We were able to confirm that circadian clock disruption reduced mitochondrial metabolism in the colon of mice. Furthermore, we demonstrated that *PER2* silencing could reduce cell proliferation and ATP production *in vitro*. This furthers our understanding of the association between the circadian clock and IBD, and opens the door for potential targeted *PER2* therapy for patients with IBD and circadian rhythm disruption.

## Materials and methods

### Mice model

All procedures used in this study followed the national guidelines and were approved by the Animal Ethics Committee of Tongji Medical College, Huazhong University of Science and Technology (ethical approval reference number: 2021S 2702). A total of 96 male C57BL/6J mice (age, 8 weeks; weight, 24–25 g) were obtained from Weitonglihua Biotechnology (Beijing, China). Following the protocol in the previous study [26], the mice were put on an 8-h time shift jet-lag schedule every 3 days that lasted for 60 days. In the last 7 days of the experiment, we induced colitis using 3% DSS (MP Biomedicals, Solon, OH, USA) that was administered in drinking water.

The mice were randomly assigned to four groups: (i) control, kept with baseline light–dark cycle (lights on at 6 a.m. and off at 6 p.m.) for the entirety of the study; (ii) jet-lagged group; (iii) control + DSS group; and (iv) jet-lagged + DSS group. Each subgroup contained 24 mice. Mice were weighed daily and fecal samples were checked for blood.

### Tissue collection

Mice were euthanized at zeitgeber time (ZT)0, ZT4, ZT8, ZT12, ZT16, and ZT20. The colon tissues were harvested, measured, and numbered separately. The distal colon was soaked in 4% formalin (cat. G1101; Servicebio, Wuhan, Hubei, China) at room temperature in preparation for hematoxylin and eosin (H&E) staining and immunohistochemistry. The remaining segments of the colon were quickly rinsed with phosphate-buffered saline (PBS) to remove lumen contents, pre-cooled at 4°C, and then stored at –80°C to be used later in quantitative polymerase chain reaction (qPCR), ATP measurement, and Western blot.

### Histology and immunohistochemistry

The colon sections fixed with 4% formalin (cat. G1101; Servicebio) were taken out, dehydrated, and embedded in paraffin. The specimens were cut into 3-µm-thick sections and stained with H&E. Two independent pathologists, who were blinded to the grouping information, assessed the pathology score using the established scoring standard [27].

For immunohistochemistry, the sections were deparaffinized in a series of xylene and ethanol solutions then placed in citrate buffer for heat-induced tissue antigen recovery. Endogenous peroxidase was put in the 3% hydrogen peroxide solution at room temperature in the dark for 25 min. To block non-specific binding, the sections were exposed to 3% bovine serum albumin for 30 min. The primary antibody Ki67 (1:200; cat. GB111499; Servicebio) was applied to sections overnight at 4°C. After washing three times in PBS, a horseradish peroxidase-conjugated secondary antibody was applied at room temperature for 50 min. Binding was visualized using 3,3'-diaminobenzidine color development. The nuclei were stained with Harris hematoxylin (cat. G1104; Servicebio) for 3 min. The sections were then dehydrated and sealed with neutral gum. Positive expressions of Ki67 on immunohistochemical indices in the colon tissues were quantified using Image-Pro Plus software (Version 6.0.0.260; Media Cybernetics, Inc., Bethesda, MD, USA).

### Transmission electron microscopy

The colon tissues were flushed with cold PBS and then cut perpendicular to the horizontal axis into 5-mm-wide blocks. They were fixed with 4% paraformaldehyde (cat. P0099; Beyotime, Shanghai, China) overnight, then soaked with osmium tetroxide, dehydrated, and embedded in resin. Tissue pieces were cut into ultrathin specimens (80 nm thick). Mitochondrial structures were visualized using a transmission electron microscope (HT7800; Hitachi, Tokyo, Japan) at ×40,000 magnification.

### Cell culture and transfection

Normal human colon epithelial cells (CCD 841 CoN) were purchased from Meisen Cell Technology (Hangzhou, Zhejiang, China) and authenticated using the short tandem repeat profiling. Cells were cultured in Dulbecco's modified eagle medium (cat. D0189; Sigma, St. Louis, MO, USA) that was supplemented with 10% fetal bovine serum (cat.10099; Gibco, Brooklyn, NY, USA) and 1% antibiotic-antimycotic (cat. 5955; Sigma) and cultured at 37°C in the humidified atmosphere with 5% (v/v) carbon dioxide. The small interference ribonucleic acids (RNAs) (siRNAs) were transfected using the Lipo3000 (cat.L300015L; Thermo Scientific, Rockford, IL, USA) according to the manufacturer’s instructions. The siRNAs were purchased by GeneChem (Shanghai, China) and the negative control siRNA was generated using Sigma (cat.SIC001). The interference sequence of siR-per2 was 5′-GGTCAAACCTCGAGACTCA-3′.

### Immunofluorescence

To observe the effect of *PER2* knockout on the expression level of Ki67 in the CCD 841 CoN cells, we cultured the same number of cells transfected with siRNA against *PER2* (siPer2) and control siRNA (siCon) on 12-well slides overnight. On the second day, 4% paraformaldehyde was used to fix the cell slides for 15 min. They were washed three times with PBS, incubated with 1% donkey serum for 1 h, and then incubated with polyclonal rabbit anti-Ki67 antibody (1:200; cat. GB111499; Servicebio) at 4°C overnight. The next day, the sections were incubated with Alexa Fluor 594 anti-rabbit secondary antibody (1:200; cat. ANT029S; AntGene, Wuhan, Hubei, China) for 1 h at room temperature and then counterstained with DAPI (cat. ANT165; AntGene). The expression of Ki67 was examined using a laser scanning confocal microscope (Si-A1; Nikon, Tokyo, Japan).

### ATP content

The colon tissues were homogenized and the CCD841 CoN cells were lysed and then centrifuged at 12,000 × *g* for 5 min. The supernatant (20 µL) was diluted in 100 µL of ATP detection reagent diluent (ATP Assay Kit, cat. S0026; Beyotime) and the luciferase activity was evaluated immediately in a single-tube luminometer. The ATP content of the samples was determined by comparison to the standard curve prepared from known amounts of ATP and normalized by protein concentration.

### Cell proliferation assay

The CCD841 CoN cell lines were transferred with siRNA then seeded in six-well plates. We added 100 µL of WST-1 reagent (WST-1 kit, cat. MK400; TaKaRa, Tokyo, Japan) to each well of cell culture medium and used a microplate reader to measure the absorbance of the samples after incubation for 2 h at 450 nm. The cells were counted at 24, 48, and 72 h after siRNA transfection. The cells in the six-well plates were treated with trypsin (cat. 25300; Gibco), centrifuged, resuspended in PBS, stained with trypan blue (cat. T8154; Sigma), and all the living cells were counted.

### Quantitative qPCR

The total RNA was isolated from mouse colon tissues and CCD 841 CoN cells with TRIzol (cat.9109; TaKaRa), then the cDNA was synthesized using Transcriptor Reverse Transcriptase (cat. RR036A; TaKaRa) and amplified using a LightCycler 480 fluorescence quantitative PCR system (Roche Diagnostics, Indianapolis, IN, USA). The relative mRNA levels were normalized against *β-ACTIN* (for CCD841 CoN cells) or glyceraldehyde-3-phosphate dehydrogenase (*Gapdh*, for mouse colon tissues) and analysed using the 2^-ΔΔCT^ method (six replicates each). The primers used for the analyses are shown in **Table 1**.

**Table 1.** Forward (F) and reverse (R) primer sequences for qRT–PCR

Gene	Sequence
Mouse Gapdh	F : 5′-AGGAGCGAGACCCCACTAACA-3′
	R : 5′-AGGGGGGCTAAGCAGTTGGT-3′
Mouse Bmal1	F : 5′-GAACGGGGAAATACGGGTGA-3′
	R : 5′-GCCTGTGACATTCTGCGAGGT-3′
Mouse Per1	F : 5′-CCCAGCTTTACCTGCAGAAG-3′
	R : 5′-ATGGTCGAAAGGAAGCCTCT-3′
Mouse Per2	F : 5′-GGGAAACACCACGAGAATGAGAT-3′
	R : 5′-GAGGGATTCTAGGCGCTTCATAG-3′
Mouse Cry1	F : 5′-TGAGGCAAGCAGACTGAATATTG-3′
	R : 5′-CCTCTGTACCGGGAAAGCTG-3′
Mouse Cry2	R : 5′-CCTCTGTACCGGGAAAGCTG-3′
	F : 5′-CTGGCGAGAAGGTAGAGTGG-3′
Mouse Clock	F : 5′-GGCGTTGTTGATTGGACTAGG-3′
	R : 5′-GAATGGAGTCTCCAACACCCA-3′
Mouse Nr1d1	F : 5′-CCGTGACCTTTCTCAGCATGA-3′
	R : 5′-CACTGTCTGGTCCTTCACGTTG-3′
Human PER2	F : 5′-GCTGGCCATCCACAAAAAGA-3′
	R : 5′-GCGAAACCGAATGGGAGAAT-3′
Human β-ACTIN	F : 5′-TCACCCACACTGTGCCCATCTACGA-3′
	R : 5′-CAGCGGAACCGCTCATTGCCAATGG-3′

### Western blot

Tissues and cells were lysed in RIPA lysis buffer (cat. P0013B; Beyotime), proteinase and phosphatase inhibitors were added to protein lysates, and proteins were concentrated using a BCA Protein Assay kit (cat.23225; Pierce, Rockford, IL, USA). The primary and secondary antibodies are provided in **Table 2**. Gapdh was used to normalize the Bmal1, Clock, Per1, Per2, Cry1, Cry2, and Rev-erbα (Nr1d1) protein fractions. Additionally, Drp1 and p-Drp1 were normalized relative to Vdac. Blot bands were visualized using the ECL reagent (cat. WP20005; Thermo Scientific). The integrated density of the blot band was measured using ImageJ software (Version1.51j8; National Institutes of Health, Bethesda, MD, USA).

**Table 2.** Antibodies for Western blot

Antibody	Company	Catalog	Specificity	Host	Dilution
Bmal1	Cell Signaling Technology	14020S	Mouse	Rabbit	1:1,000
Clock	Cell Signaling Technology	5157s	Mouse	Rabbit	1:1,000
Nr1d1	Cell Signaling Technology	13418S	Mouse	Rabbit	1:1,000
Cry1	Abcam	Ab171860	Mouse	Rabbit	1:1,000
Cry2	Proteintech	13997-1-AP	Mouse	Rabbit	1:500
Per1	Proteintech	13463-1-AP	Mouse	Rabbit	1:500
Per2	ABclonal	A5107	Mouse	Rabbit	1:500
Per2	Abcam	ab179813	Human	Rabbit	1:1,000
DRP1	Cell Signaling Technology	8570S	Mouse and Human	Rabbit	1:1,000
P-DRP1	Cell Signaling Technology	4867S	Mouse and Human	Rabbit	1:1,000
Gapdh	Cell Signaling Technology	5174S	Mouse and Human	Mouse	1:2000
Vdac	Abcam	ab14734	Mouse and Human	Mouse	1:1,000
anti-rabbit	Cell Signaling Technology	7,076		Goat	1:3,000
anti-mouse	Cell Signaling Technology	7,074		Horse	1:3,000

### Statistical analyses

The statistical analyses and data presentation were performed using GraphPad Prism (Version9.2.0; GraphPad Software, Inc., San Diego, CA, USA). The data were analysed using unpaired two-tailed Student's *t*-tests and one-way analysis of variance (ANOVA) to determine significance. The results were presented as mean  ± standard deviation (SD). A *P-*value of <0.05 was considered statistically significant.

## Results

### Jet lag disrupted the rhythmicity of core clock genes in the Colon tissue

**Figure 1.**
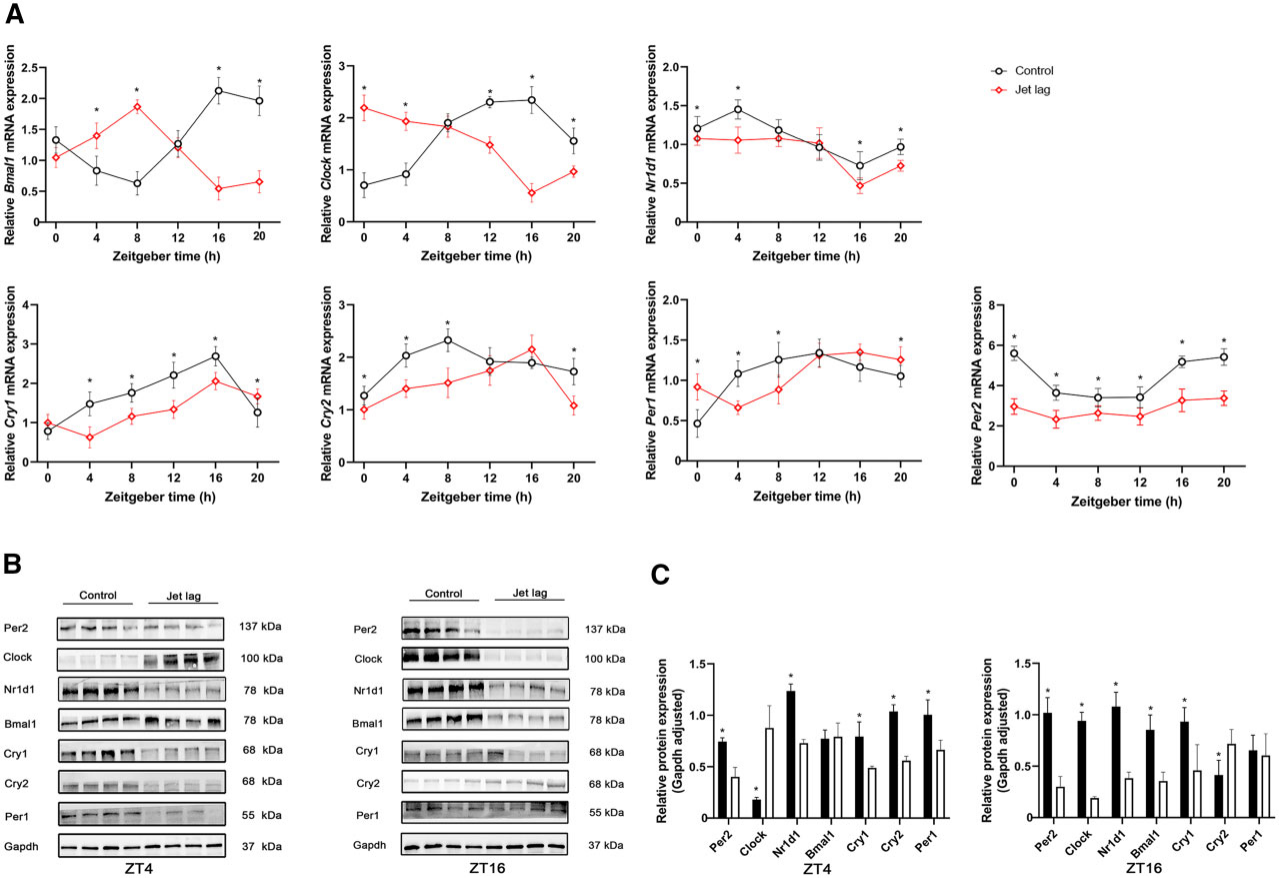
Jet lag disrupted the rhythmicity of circadian clock genes. The total mRNA was isolated from the colon tissue and qPCR was carried out using different primers. Gapdh was used as a normalized control. (A) The qPCR assays of the core clock genes in mice in the jet-lag and control groups at different time points (six mice per group). (B) Western blot analysis of the core clock genes at zeitgeber time (ZT)4 and ZT16 (six mice per group). (C) The relative expression of Bmal1, Clock, Per1, Per2, Cry1, Cry2, and Nr1d1 normalized with Gapdh (six mice per group). Presented data are expressed as mean ± SD. *P < 0.05 vs control mice at individual time points (*t*-test).

The core circadian clock genes *Bmal1*, *Clock*, *Per1*, *Per2*, *Cry1*, *Cry2*, and *Nr1d1* in mice were detected using qPCR. The transcription of these genes oscillated in the whole colon tissue indicating clock rhythmicity at the molecular level. After a jet-lag schedule, the clock genes still showed rhythmicity, but the rhythmicity differed from that of mice in the control group (Figure 1A). Notably, *Per2* mRNA expression was decreased at all six points in time. The *Per2* gene has been shown to play an important role in regulating cell proliferation, longevity, and energy metabolism [28–30]. The protein expression levels of core clock genes were measured at ZT4 and ZT16 (Figure 1B and C). We found that the protein expression of Bmal1 was not parallel with the mRNA changes at ZT4. Many factors, which may be related to our insufficient sample size or the post-transcriptional modification of the protein, might have affected the amount of protein expression. The changes of other core clock genes in the protein expression level were consistent with those in the mRNA expression level. These show that circadian rhythm changes disrupted the rhythm of clock genes in the colon of mice.

### Circadian rhythm disruption caused mitochondrial dysfunction

**Figure 2.**
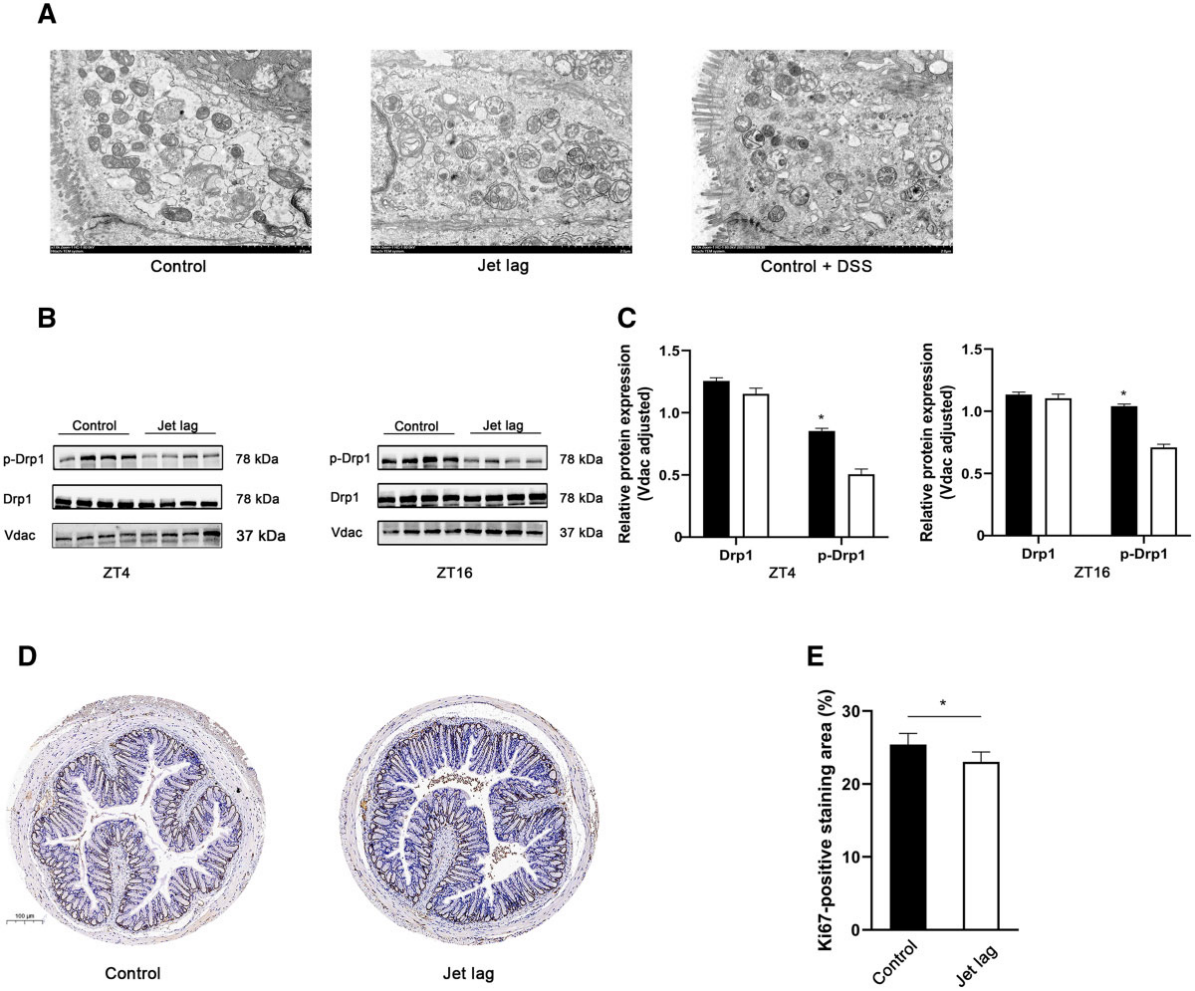
Mitochondrial alterations after jet lag. (A) Representative mitochondrial morphological changes were detected using transmission electron microscopy (six mice per group). (B) Western blot analysis of Drp1 and p-Drp1 at ZT4 and ZT16 (six mice per group). (C) The relative expression of Drp1 and p-Drp1 normalized with Vdac (six mice per group). (D) Photomicrograph of mice colon tissue immunohistochemical analysis for Ki67. (E) Qualification of Ki67-positive staining area (six mice per group). The values are expressed as mean ± SD. *P < 0.05 vs control mice at individual time points (*t*-test). ZT, zeitgeber time; DDS, dextran sodium sulfate.

Circadian rhythm plays a crucial role in mitochondrial energy metabolism [7]. We explored the effect of jet lag on mitochondrial morphology and function. Jet lag destroyed mitochondrial morphology resulting in increased mitochondrial fragmentation (decreased mitochondrial membrane integrity, ambiguous myofilaments, and completely unstructured cristae). In addition, we observed fragmented mitochondria in the colon tissue of mice with DSS-induced colitis, which means that mitochondrial morphology and function are impaired in colitis (Figure 2A).

Schmitt *et al.* [18] demonstrated that rhythmic phosphorylation of DRP1 at Serine 637 leads to circadian ATP production. Therefore, we determined the expression levels of Drp1 and p-Drp1. In Figure 2B andC, no significant difference was detected in Drp1 expression between mice in the control and the jet-lagged groups, which is consistent with what previous studies have found. However, the protein expression of p-Drp1 was decreased at ZT4 and ZT16 in the jet-lagged groups. These findings indicate that circadian rhythm disruption could affect both the morphology and biological function of mitochondria, hence reducing ATP production in the colon of mice. Intestinal epithelial cells require constant proliferation to maintain self-renewal and the integrity of the intestinal epithelial barrier. Because ATP is closely related to cell cycle and proliferation, we used immunohistochemistry to detect the expression of Ki67 protein in the intestinal tract of mice. Ki67 is a marker of cell proliferation and is expressed throughout the cell proliferation cycle [31, 32]. As expected, Ki67 expression in the colon was significantly reduced in the jet-lagged group (Figure 2D and E).

### Circadian rhythm disruption worsened DSS-induced colitis

**Figure 3.**
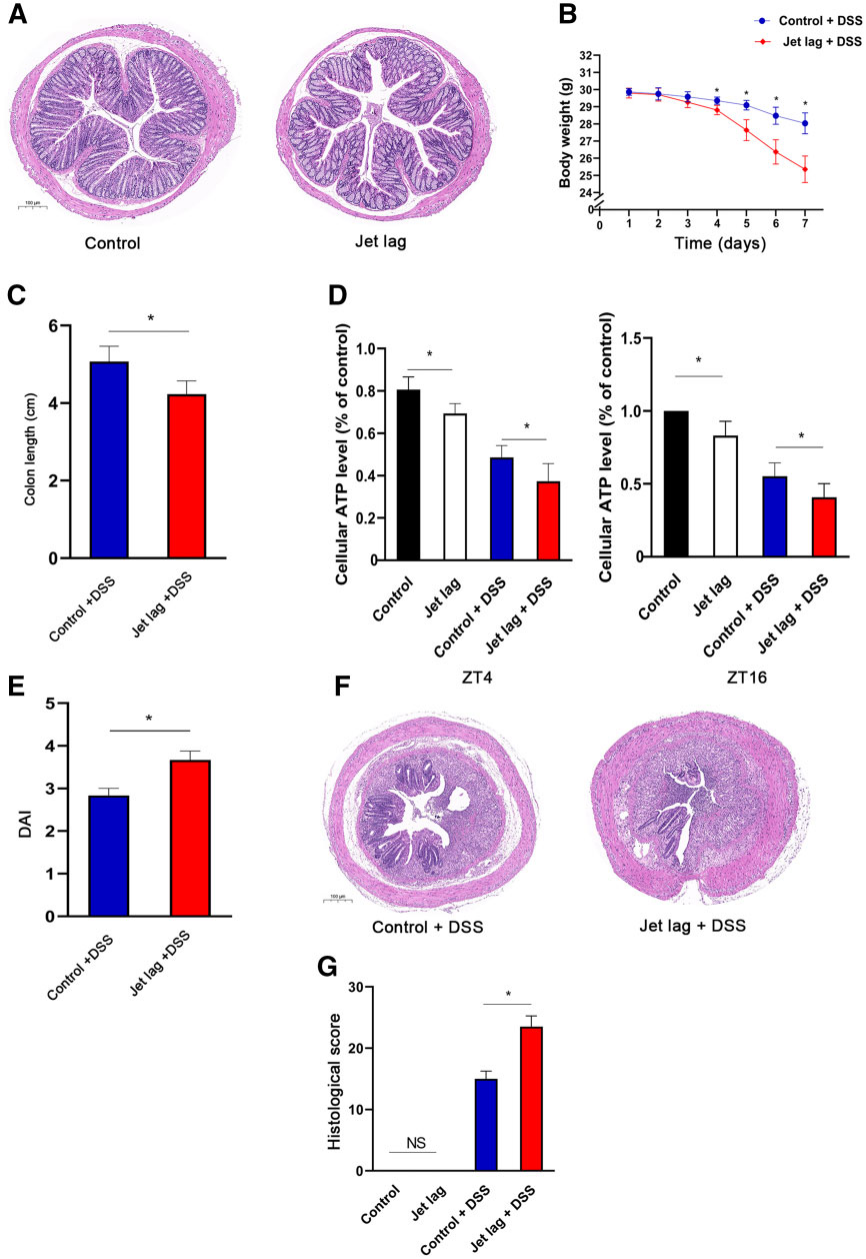
Jet lag aggravated DSS-induced colitis. Chronic circadian desynchronization alone did not have significant effects on body weight or colon histology. (A) Representative images of H&E-stained colon tissue sections revealed no significant difference in structure or lymphocyte infiltration between the two groups (six mice per group). (B) From the fourth day after DSS treatment, body weight was significantly decreased in the jet lag + DSS group than in the control + DSS group (six mice per group, *t*-test). (C) The colon length was significantly shorter in the jet lag + DSS group than in the control + DSS group (six mice per group; *t*-test). (D) The ATP level was significantly lower in the jet-lag group than in the control group, and there was a further decrease in the jet lag + DSS group compared with the ATP level in the control + DSS group at ZT4 and ZT16 (six mice per group; one-way ANOVA). (E) The disease activity index (DAI) score was significantly higher in the jet lag + DSS mice group than in control + DSS mice group (six mice per group; *t*-test). (F) DSS-induced colitis was exacerbated in jet-lagged mice, as evidenced by more extensive destruction of the mucosal layer and mucosal ulceration. (G) The histological score was significantly higher in jet lag + DSS mice group than in the control + DSS mice group and there was no significant difference between the control and the jet-lag groups (six mice per group; one-way ANOVA). Data are expressed as mean ± SD. *P < 0.05. ZT, zeitgeber time; DDS, dextran sodium sulfate; H&E, hematoxylin eosin; DAI, disease activity index; NS, not significant.

Chronic disruption of the circadian rhythm without inducing colitis did not significantly influence the weight of the mice or colon histology. The weights were similar between the mice in the control and the jet-lag groups. No significant differences were observed in the structure or lymphocyte infiltration in the H&E-stained tissue of both groups (Figures 3A).

Mice in the jet lag + DSS group had lower body weight on Day 4 compared with the mice in the control + DSS group (Figure 3B). The colon length was significantly shorter in the jet lag + DSS group compared with that in the control + DSS group (Figure 3C). The ATP level was lower in the jet lag + DSS group than in the control + DSS group (Figure 3D). The disease activity index scores were substantially higher in the jet lag + DSS group than in the control + DSS group (Figure 3E). The histopathology scoring of H&E-stained sections revealed significantly greater colon structural damage evidenced by mucosal injury, glandular atrophy, and inflammatory cell infiltration in the jet lag + DSS group than in the control + DSS group (Figure 3F and G). Together, these suggest that disrupting the circadian rhythm worsens colitis in mice and decreases mitochondrial energy metabolism in the colon.

### *PER2* silencing reduced ATP production and proliferation of CCD 841 CoN cells *in vitro*

*In vitro* experiments showed that circadian rhythm disruption caused the downregulation of *Per2* expression and the ATP level. Given that *Per2* can reduce ATP production by reducing Drp1 phosphorylation at ser637 and the level of ATP is closely related to cell proliferation, a poor proliferation of intestinal epithelial cells is the key factor in the delayed recovery of patients with ulcerative colitis.

**Figure 4.**
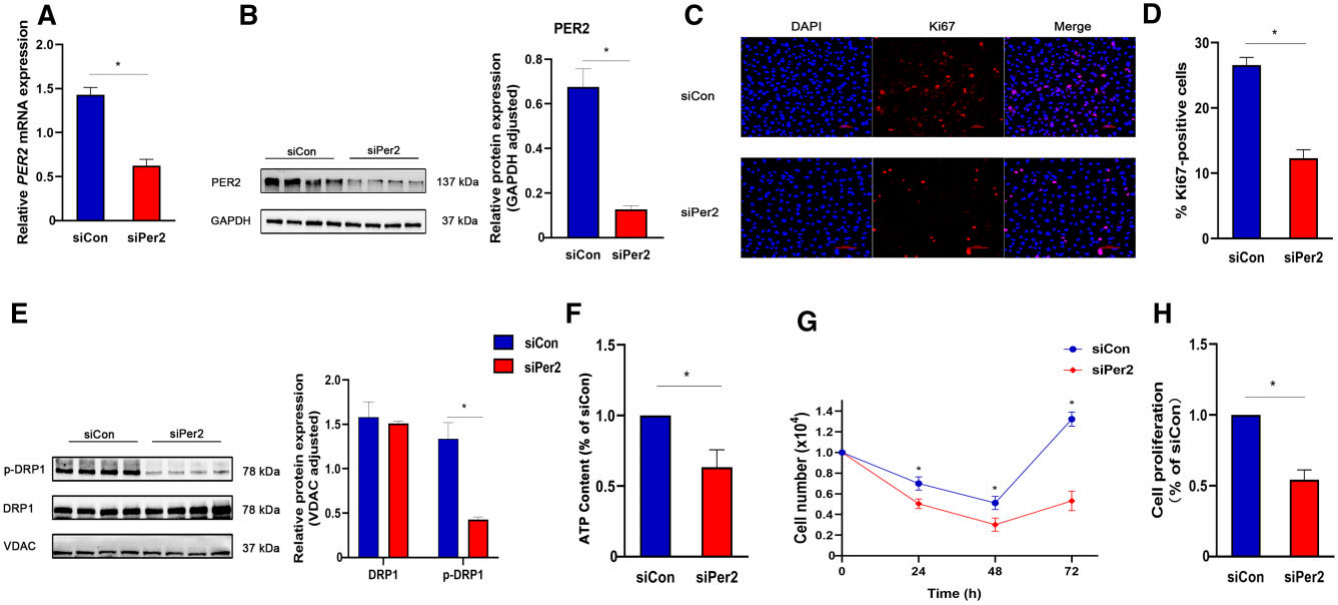
*PER2* silencing inhibited proliferation and ATP production of CCD 841 CoN cells *in vitro*. (A) qPCR was used to detect the expression of *PER2* mRNA in CCD 841 CoN cells after transfection with siRNA (*n* = 6). (B) Protein expressions of PER2 after transfection with siRNA (*n* = 6). (C) Immunofluorescence images of Ki67 expression after siRNA transfection with CCD 841 CoN cells. Red fluorescence indicated Ki67, DAPI stained nuclei (*n* = 6). (D) Quantification of Ki67-positive cells (*n* = 6). (E) Protein expressions of DRP1 and p-DRP1 after transfection with siRNA (*n* = 6). (F) The ATP content of CCD 841 CoN cells 72 h after transfection with siRNA (*n* = 6). (G) Counting of CCD 841 CoN cells 72 h after transfection with siRNA (*n* = 6). (H) Proliferative activity of CCD 841 CoN cells measured using WST-1 assay 72 h after transfection with siRNA (*n* = 6). Presented values are mean ± SD. *P < 0.05 vs siCon CCD 841 CoN cells (*t*-test).

We investigated whether *PER2* silencing in CCD 841 CoN cells, which are normal colon epithelial cells, could inhibit ATP production and cell proliferation. CCD 841 CoN cells were transfected with the siRNA against *PER2* (siPer2) or the control siRNA (siCon). The knock-down level was verified through qPCR and Western blot (Figure 4A and B). We first cultured an equal number of siRNA-transfected cells on 24-well plates and observed the expression of Ki67 in CCD 841 CoN cells using immunofluorescence. Compared with siCon, *PER2* silencing significantly reduced the expression of Ki67 (Figure 4C and D). Furthermore, *PER2* silencing markedly reduced the expression level of p-DRP1 (Figure 4E), which is consistent with the results of Schmitt *et al*. [7] and our *in vitro* study. We also counted the cells after transfection. Because cell proliferation is relatively fast in the medium containing serum and this may obscure the effect of *PER2* silencing, we used a serum-free medium for the first 2 days after siRNA transfection. During the first 2 days, the number of cells continued to decrease in the siPer2 and siCon groups. Subsequently, we added 5% fetal bovine serum to the medium. The ATP level was lower in the siPer2 group than in the siCon group (Figure 4F). The number of cells was lower in the siPer2 group compared with that in the siCon group (Figure 4G). WST-1 assay confirmed the lower proliferative activity of cells in the siPer2 group 72 h after transfection compared with that in the siCon group (Figure 4H). These data suggest that *PER2* silencing reduced ATP production and cell proliferation.

**Figure:**
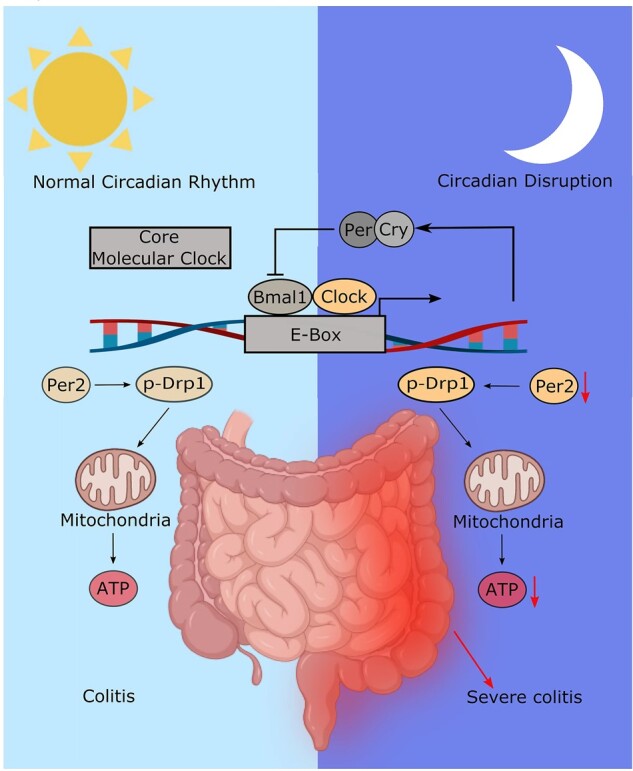


## Discussion

Our study aimed to investigate the effects of circadian rhythm disruption on DSS-induced colitis in mice. Compared with the control group, the oscillation mode of the core circadian clock genes (*Bmal1*, *Clock*, *Per*, *Cry*, and *Nr1d1*) of jet-lagged mice changed. The disruption of the circadian rhythm caused changes in the morphology of the mitochondria of mice colon, reduced ATP production and cell proliferation, and aggravated colitis in mice.

As the pace of modern life accelerates, more and more people have their circadian rhythm disrupted due to jet lag caused by factors such as long-distance travel and shift work. Studies have found that humans are more sensitive to night light than was initially thought [33], so the use of electronic products before going to bed may also disrupt the circadian rhythm. Understanding the underlying molecular mechanisms between circadian rhythm disruption and IBD is of great significance in the treatment of patients with IBD. To understand these mechanisms, we established a jet-lagged mouse model and induced colitis using DSS. Jet lag changed the colon core clock genes oscillation and reduced *Per2* expression at six time points. Because Per2 can affect the expression of p-Drp1, which is positively correlated with the production of ATP [18], levels of p-Drp1 and ATP were lower in the jet-lagged mice compared with the control mice. Additionally, using TEM, we observed the mitochondrial morphological changes including swelling, reduction of inner cristae, and destruction of the integrity of the mitochondrial membrane. These all suggest increased mitochondrial fragmentation and apoptosis. Similar to our research, Shah *et al*. [34] confirmed that mitochondrial dysfunction occurs in the early stage of colitis and is an early marker for colitis. Smith *et al*. [35] demonstrated the presence of mitochondrial dysfunction in an induced colitis rodent model and human IBD. Jet lag resulted in morphological changes in mitochondria and reduced biological functions, both of which reduced ATP production in the mice colon. Therefore, jet lag may aggravate DSS-induced colitis in mice through mitochondrial injury.

The intestinal epithelium is in the dense microbial environment and constitutes the intestinal surface barrier. In most mammals, intestinal epithelial cells need to be renewed every 2–5 days to maintain their barrier function and adapt to the intestinal microenvironment [36]. Intestinal stem cells (ISCs) with G protein-coupled receptor 5^+^ (Lgr5^+^) are considered to be the most rapidly proliferating ISCs, which continuously proliferate and differentiate into intestinal epithelial cells [37]. More studies have confirmed that mitochondria determine the homeostasis of intestinal epithelium cells and maintain the ISCs [38, 39]. The process of cell proliferation requires ATP. We found that the expression of Ki67 in the colon of mice was decreased and because it is a proliferation marker, this reflects the reduced ability of the colon epithelium to proliferate, maintain its integrity, and resist external injury. Because *in vitro* experiments showed that *Per2* decreased at six time points, we silenced *PER2* in the CCD 841 CoN cells and detected decreased p-DRP1, ATP, and cell proliferation levels.

Presently, the association between circadian rhythm disruption and IBD remains elusive. It is generally believed that there is a tight relationship between the circadian rhythm and the immune system. Circadian rhythm disruption could aggravate IBD by affecting the immune system, but there are few studies on the effect of circadian clock genes on intestinal epithelial cells. Wang *et al*. [19] reported that Nr1d1 inactivates NLRP3 inflammasomes by repressing the production of NF-κB and NLRP3 at the transcription level, thereby reducing inflammation. Yu *et al*. [40] reported that Type 17 helper T-cells are regulated by the circadian clock genes and are involved in the development of IBD. Stokes *et al*. [13] confirmed that the *Bmal1* gene could promote the 24-h rhythmic production of intestinal epithelial cells, thereby reducing colitis caused by radiation. Our study further sheds light on the role of circadian clock disturbances in the occurrence and development of IBD.

This study has certain limitations. Only the jet-lagged mice model was used for the *in vivo* experiment, but no clinical data were collected. Second, Lgr5^+^ ISCs were not used in the *in vitro* experiments. Further studies of the relationship between circadian disruption and ulcerative colitis severity require larger sample sizes, longer follow-up, and use of a *Per2* knockout animal model. In addition, we did not discuss the possible mechanism by which jet lag causes mitochondrial morphological changes.

In summary, our research provides crucial evidence to confirm that circadian rhythm disruption aggravates DSS-induced colitis in mice and proves that inhibiting *Per2* expression can reduce ATP production and cell proliferation. Our findings potentially provide a new therapeutic target in patients with IBD and circadian rhythm disruption.

## Authors’ Contributions

Y.D.C. conceived and designed the project. R.F.Z., G.Z., and F.M.L. collected the data. J.R.L., M.Y.X., D.G., Q.L.Z., and S.L. analysed and interpreted the data. All authors read and approved the final manuscript.

## Funding

This work is supported by National Key R&D Program of China [No. 2018YFC0114600] and National Natural Science Foundation of China [No. 82170547 and No. 81873558].

## Conflict of Interest

All authors declare no conflict of interest.
